# Design, set-up and utility of the UK facioscapulohumeral muscular dystrophy patient registry

**DOI:** 10.1007/s00415-016-8132-1

**Published:** 2016-05-09

**Authors:** Teresinha Evangelista, Libby Wood, Roberto Fernandez-Torron, Maggie Williams, Debbie Smith, Peter Lunt, Judith Hudson, Fiona Norwood, Richard Orrell, Tracey Willis, David Hilton-Jones, Karen Rafferty, Michela Guglieri, Hanns Lochmüller

**Affiliations:** John Walton Muscular Dystrophy Research Centre, Institute of Genetic Medicine, Newcastle University, Central Parkway, Newcastle upon Tyne, NE1 3BZ UK; Neurology Department, Donostia University Hospital, Donostia-San Sebastian, Spain; Neuromuscular Area, Biodonostia Health Research Institute, Donostia-San Sebastian, Spain; Bristol Genetics Laboratory, Southmead Hospital, North Bristol NHS Trust, Bristol, UK; Department of Neurology, King’s College Hospital, London, UK; The Robert Jones and Agnes Hunt Orthopaedic Hospital, Oswestry, UK; Department of Clinical Neurology, John Radcliffe Hospital, Oxford, UK

**Keywords:** Registries, FSHD, Clinical trials, Minimal dataset, Data sharing, Rare diseases

## Abstract

Facioscapulohumeral dystrophy (FSHD) is a rare inherited neuromuscular disease estimated to affect 1/15,000 people. Through basic research, remarkable progress has been made towards the development of targeted therapies. Patient identification, through registries or other means is essential for trial-readiness. The UK FSHD Patient Registry is a patient initiated registry that collects standardised and internationally agreed dataset of self-reported clinical details combined with professionally verified genetic information. It includes four additional questionnaires to capture patient reported outcomes related to pain, quality of life and scapular fixation. Between 2013 and 2015, 518 patients registered 243 males, 241 females with a mean age of 47.8 years. Most of the patients have FSHD type 1 (91.7 %), and weakness of the facial (59.2 %) was the most prevalent symptom at onset, followed by shoulder-girdle muscles (53.3 %) and distal (22.45 %) or proximal lower limb weakness (14.8 %). 85.57 % patients were ambulant or ambulant with assistance at the time of registration, 7.9 % report respiratory insufficiency. The registry has demonstrated utility with the recruitment of patients for a natural history study of infantile onset FSHD, and the longitudinal analysis of patient-related outcomes will provide much-needed baseline information to power future trials. The internationally agreed core dataset enables national registries to participate in a “Global FSHD registry”. We suggest that the registry’s ability to interoperate with other large datasets will be instrumental for sharing and exploiting data globally.

**Electronic supplementary material:**

The online version of this article (doi:10.1007/s00415-016-8132-1) contains supplementary material, which is available to authorized users.

## Introduction

FSHD one of the three most frequent neuromuscular disorders [1] with an estimated prevalence of 1/15,000–1/20,000 [2, 3]. There is currently no treatment to slow down, reverse or cure the symptoms of FSHD. Symptoms usually start around the second decade and are characterised by progressive and sometimes markedly asymmetric muscle weakness, affecting the facial, scapular or humeral muscles. Later on, muscle weakness may involve the abdominal muscles and the muscles of the legs and feet [4]. FSHD is an autosomal dominant disease associated in the majority of patients (95 %) with a contraction of a polymorphic repeat D4Z4 on chromosome 4qA (FSHD1) [5–7]. In 5 % of FSHD patients there is a normal or intermediate length of the D4Z4 macrosatellite. Recently, mutations in the *SMCHD1* gene (structural maintenance of chromosomes flexible hinge domain containing 1) have been described in patients with an FSHD phenotype and normal D4Z4 length, now described as FSHD2 [8]. A common, digenic mechanism has been proposed for FSHD 1 and 2, involving abnormal epigenetic regulation of the D4Z4 macrosatellite on 4qA in both forms of the disease. In FSHD 1 this is due to a deletion in the heterochromatin (D4Z4) and in FSHD 2 is associated with loss-of-function mutations in *SMCHD1* a gene responsible for the maintenance of heterochromatin. *SMCHD1* mutations will result in hypomethylation of D4Z4. If a permissive 4qA allele is present, both mechanisms lead to an increased expression of the deleterious retrogene *DUX4* [4].

This increased understanding of the genetic mechanism of FSHD has led to an increase in therapeutic strategies with a number of pharmaceutical companies exploring programmes for FSHD. However, there are many challenges in the development of therapies and moving them from bench to bedside [9]. These include the recruitment of sufficient numbers of well-characterised patients to ensure meaningful and statistically significant results. Registries provide a tool to help overcome this challenge by engaging well-defined cohorts of patients interested in participating in research [10]. Patient registries also have the potential to collect data on disease burden and treatment interventions with patient reported outcomes currently being used in 14 % of clinical trials [11]. The UK FSHD patient registry (http://www.fshd-registry.org/uk), launched in May 2013, provides an online portal in order to collect these outcomes.

The registry is funded by Muscular Dystrophy UK (MDUK) and was developed under the umbrella of the global neuromuscular network TREAT-NMD (http://www.treat-nmd.eu/) [12]. Here we describe the setup, progress and utility of the registry together with the demographic details of the patients registered in its first 2 years (May 2013–May 2015). We further discuss the accuracy and completeness of the data.

## Methods

### Design and set up

#### Dataset

The UK FSHD registry collects all mandatory and highly encouraged items outlined in this dataset for both patients with FSHD1 and FSHD2 and is coordinated from the John Walton Muscular Dystrophy Research Centre, Newcastle, UK. The dataset comprises all the items identified at the 171st ENMC (European Neuromuscular Centre) workshop on the care and management of FSHD [13]. This is a minimal dataset designed to incorporate the features that may define inclusion/exclusion criteria for clinical trials. The mandatory items help to characterise the demographics, genetics, motor function and age of onset of the disease. The highly encouraged data includes additional aspects that may help to better characterise the population like eye and hearing involvement, respiratory status and family history (Table 1). The registry goes beyond this core dataset to collect additional information of interest, identified by the UK researchers involved and the patient community. After consultation with professional and patient groups, outcomes relating to pain, quality of life and scapular fixation were included. The outcomes used include validated questionnaires; the McGill short form pain questionnaire (MPQ) [14] and the Individualised Neuromuscular Quality of Life Questionnaire (INQoL) [15]. We also devised a universal pain assessment tool (FSHD pain questionnaire, supplementary online material) and a questionnaire about scapular fixation (supplementary online material). These questionnaires have been developed specifically for the registry and require further validation.

**Table 1:** Items defined in the internationally agreed core dataset for FSHD registries (full questionnaire in online supplementary material) [16]

Mandatory items	Highly encouraged items
Personal data (name, date of birth, address, phone, email) Genetic test result (FSHD1, FSHD2) Clinical data (facial weakness, shoulder weakness, foot weakness, hip girdle weakness) Onset of muscle weakness Best current motor function (ambulatory, non-ambulatory) Wheelchair use	Ventilation (non-invasive, invasive) Age of onset for selected FSHD symptoms Retinal vascular disease attributable to FSHD Hearing loss Scapular fixation (one shoulder, both shoulders) Pregnancy (females only) Family history Ethnic origin Other registry involvement

#### Governance and data access

The registry Steering Committee has 13 members including clinical and genetic experts as well as representatives from patient organisations and patients themselves (full details in acknowledgments). The committee has input on the strategic direction of the registry, acts as a data access committee and ensures that the registry acts in the best interests of patients. Terms of reference and standard operating procedures have been developed for the committee (available in supplementary online material). The registry is managed and maintained by a registry curator, Libby Wood who has been responsible for the registries development since 2012, with previous work in establishing the steering committee being carried out by Karen Rafferty from 2011.

The registry acts as a trusted intermediary between researchers (academic or commercial) and patients themselves. A researcher may request aggregate de-identified data through a proposal to the steering committee. The committee do not provide ethical and scientific critique but requires evidence these have been carried out by the appropriate bodies if necessary. The committee evaluates if providing the data will benefit the patients registered. A vote takes place within 2 weeks, with data being provided within 4 weeks of a positive outcome. The same evaluation process is required before information is disseminated to help recruitment. Researchers do not receive direct access to patients or to personal identifiable information. The registry services are free of charge to academics and charitable organisations but a fee structure is in place for commercial requests (available in online supplementary material).

#### Data collection

Potential participants hear about the registry through patient support and advocacy groups (notably FSHD Support UK and Muscular Dystrophy UK), who provide information at conferences, on websites and in newsletters. In addition, clinicians received leaflets which are distributed at clinical appointments. Information about the registry is also included on all diagnostic genetic reports positive for FSHD issued by UK genetic laboratories. In all cases the participants initiate registration themselves online creating a personal account to start registration.

The UK FSHD Registry (http://www.fshd-registry.org/uk) uses an advanced combined reporting system allowing patient and clinician reported data to be united through a single online portal. The information in the registry is stored securely on a dedicated server at the Medical Centre of Ludwig-Maximilians-Universität in Munich with limited access by named registry personnel only. The registry software was developed by the operators to enable secure, web-based collection of patient data. It is based on an open-source stack including Java EE and the PostgreSQL database server.

The registry is designed to allow the majority of the information to be provided by the patients themselves, after consenting online. Age appropriate information and consent forms are available, enabling parents or guardians to consent on behalf of those under 18 years old (available in online supplementary material). The consent form allows for future contact, for both communication and research, to be made and for the additional data to be entered by a nominated medical professional. Participants are able to withdraw their consent at any time. All data remain the property of the patient themselves and can be withdrawn at any time.

The patient, as part of the registration process, will nominate a neuromuscular specialist from a pre-defined list who, using a separate online account, can confirm the genetic details, add missing clinical information and check for any inaccuracies in the patient data. Data from the patient and clinician are combined in a single database. In addition, genetic confirmation can be provided by the diagnostic laboratories in the UK. Since 2015 Bristol Genetics Laboratory (Bristol) is the only laboratory in the UK providing the clinical diagnostic test for FSHD; however, historical genetic data can be provided by the Northern Genetics service (Newcastle) and All Wales Medical Genetic Service (Cardiff). The diagnosis for FSHD2 has been available in Bristol since January 2014.

Participants are encouraged to update details annually allowing for the collection of longitudinal data. Where access to the internet is not available an alternative paper version of the registry questionnaire and consent procedures are provided.

#### Ethics approval

The registry has received full ethical (Newcastle and North Tyneside 113/NE/0048, February 2013), management (Newcastle upon Tyne Hospitals Trust R&D 6573, February 2013) and data protection (Caldicott February 2013) approvals for conducting these activities in the UK.

#### Data analysis

For purposes of analysis of the current data we have considered FSHD1 when the genetic report states that the patient has a contraction of the D4Z4 macrosatellite on chromosome 4qA of less than 38 kb, and FSHD2 if the genetic result confirms a *SMCHD1* mutation with a permissive 4qA allele or hypomethylation. When this data was not available we used the patient self-reported diagnosis of FSHD1 or FSHD2. All statistics discussed here are descriptive and presented as percentages of the whole cohort (*n* = 518), FSHD1 cohort (*n* = 475) or FSHD 2 cohort (*n* = 9) unless otherwise stated. All means are presented ± the standard deviation.

## Results

### Progress of data collection and other initiatives

The registry was launched at a patient information day in the UK, and presented to around 90 delegates. Two months after the launch 225 people had registered. Over the first 2 years (25 months) period an average of 21 people have registered per month. Thirty-four doctors have agreed to take part in the registry and provide genetic data. Two hundred and twenty-six patients have selected a clinician to provide additional details. Of the 518 people registered, 260 have logged into update their data, 121 have not logged in after an annual reminder and 137 have not yet been registered for 12 months (Online Supplementary Material Fig. 1: Rate of patient enrolment; May 2013 to May 2015.)

The additional outcomes (pain, scapular fixation and quality of life) have been completed by the majority of people. Of the 518 people registered in total 425 (82 %) provided answers to InQoL, 479 (92 %) answered the McGill pain questionnaire and 468 (90 %) answered the FSHD specific pain questionnaire. Within the core questionnaire 46 people report having had scapular fixation, of which 40 have completed the additional questionnaire.

In its first 2 years, the registry has helped recruitment into an international natural history study for infantile onset FSHD “A multicentre collaborative study on the clinical features, expression profiling, and quality of life of infantile onset facioscapulohumeral muscular dystrophy.” (ClinicalTrials.gov Identifier; NCT01437345). Interrogation of the registry database identified 33 patients meeting some or all of the inclusion criteria. All patients were contacted and five were included in the study. This made up 42 % of the total participant requirement for the UK study site (John Walton Muscular Dystrophy Research Centre); the remaining participants were identified from the local clinic population.

### Demographics

**Fig. 1:**
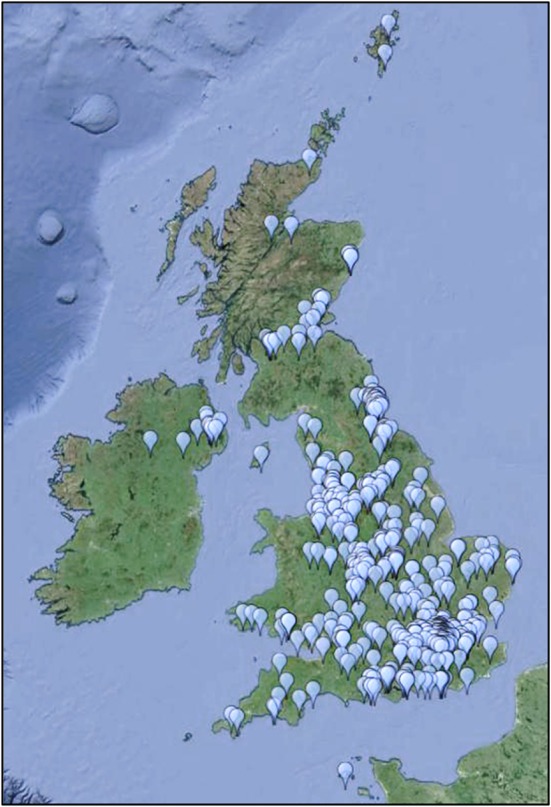
Distribution of patients across the UK, each pin represents an individual registered

Between May 2013 and May 2015 a total of 518 patients registered with the UK FSHD Patient Registry, an average of 21 patients per month from all across the UK (Fig. 1). Most patients live in the south of England with the major clustering around London. Other clusters surround the areas of Liverpool/Manchester, Newcastle and Edinburgh. These clusters represent most probably a higher population density and the existence of centres with neuromuscular specialists. Of these 475 reported being affected by FSHD 1 (91.7 %), 9 reported FSHD2 (1.7 %) and 34 (6.6 %) reported that they had a yet to be confirmed diagnosis. Excluding the patients with unconfirmed diagnosis, 98.14 % of patients were reported as FSHD1 and 1.9 % as FSHD2. Those with an unknown diagnosis have been excluded from further analysis. All data mentioned has been reported by patient themselves with the exception of genetic confirmation which has been provided by the treating neuromuscular specialist or central diagnostic lab.

The self-reported diagnosis was checked against the result of the genetic testing when available. FSHD1 is genetically confirmed in 307 cases, 286 of which had identified as having FSHD1, three misreported having FSHD2, and 18 reported an unknown diagnosis or left this question blank. We have a genetically confirmed diagnosis for two FSHD2 patients, matching the self-report in both cases.

**Fig. 2:**
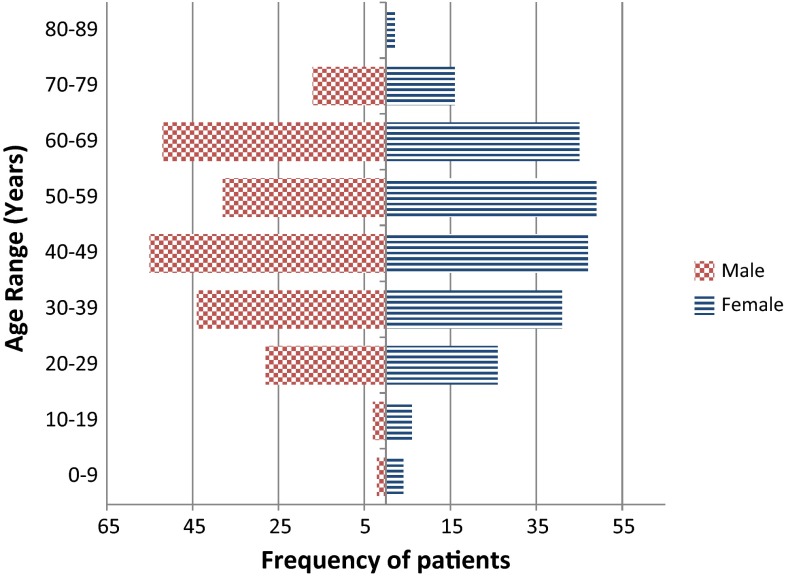
Distribution of age and gender within the UK FSHD patient registry

The mean age of all patients included in the registry is 47.82 ± 16.08; (range 6–83) years. There are 243 (50.20 %) males and 241 (49.79 %) females registered with a slight non-significant predominance of females in the younger age groups (0–19). Considering only the 475 FSHD1 patients, there is also an even distribution across genders (239 Males, 236 Females) and the current age ranges from 4 to 83 years old (mean 47.7 ± 16.13), 60.21 % (286) of patients are between the ages of 40 and 70 years old (Fig. 2). The age range of the nine FSHD2 patients is from 19 to 67 with a mean of 50 (±13.85) years, with an even distribution between genders (5 males; 4 females). Detailed family history is not collected; however, the majority of patients are aware of at least one additional family member being affected (father 79, mother 131, sibling 145, and other family member 157) (Table in online supplementary material).

The ethnic origin of all FSHD patients is predominantly Caucasian, in FSHD1 this makes up to 89.9 % (427) of those registered, [Asian 20 (4.2 %), black African 1 (0.2 %), mixed 4 (0.84 %), declined 13 (2.7 %), other 10 (2.1 %)] and in FSHD2 8 of the 9 (88.9 %) patients reported Caucasian ethnicity with the remaining patients of unknown ethnic origin. Comparison with the ethnic distribution in the 2011 Census the different ethnic groups are evenly distributed apart from the black African group that are at a lower percentage than the general population, although applying a two-sample test of proportions the difference is non-significant (Table 2).

**Table 2:** Comparison between different ethnic groups

Ethnic group	Census 2011 (%)	Registry (%)
Caucasian	87.10	89.90
Asian	6.90	4.20
Black	3.00	0.20
Mixed	2.00	0.84
Other	1	2.10
Declined		2.70

UK general population vs patients in the registry

### Clinical data

Fourteen records were excluded from this analysis, 12 FSHD1 and two FSHD2, these records have been excluded due to incomplete patient reported data. Excluded records had one or more of the following data items missing; to current motor function, hearing loss, wheelchair use, facial weakness, foot drop, hip girdle weakness and periscapular weakness. The analysis of the results showed that of the FSHD1 patients 82.5 % (*n* = 382) are ambulant although 45.1 % (*n* = 171) of these report that they require some assistance (e.g., using a cane or wheelchair part time). All of the FSHD2 patients remain ambulant with three requiring assistance. Muscle weakness is often reported to begin in multiple areas most commonly including facial muscles (59.18 %), with shoulder girdle weakness reported first in 53.33 % of cases, foot dorsiflexion weakness in 22.45 % and hip-girdle in 14.79 % (Table 3).

**Table 3:** Clinical summary of participants reporting a diagnosis of FSHD1 in the UK FSHD patient registry

	FSHD 1 (*n* = 463^a^)	Genetically confirmed (*n* = 298)	% of genetically confirmed reporting symptom (%)
Hearing loss			
Yes/no (unknown)	80/345 (38)	54/222 (22)	18.12
Retinal vascular disease^b^			
Yes/no (unknown)	10/371 (65)	9/230 (59)	3.02
Current motor function			
Ambulant	211	143	47.99
Ambulant with assistance	171	112	37.58
Non-ambulant	81	43	14.43
Wheelchair use			
Full-time	82	45	15.10
Part-time	103	65	21.82
None	278	188	63.09
Facial weakness	347	214	71.81
Scapular weakness	428	270	90.60
Hip girdle weakness	346	214	71.81
Foot drop	341	215	72.15

^a^12 records excluded for missing or incomplete patient reported data

^b^An additional 17 participants did not respond to the question about retinal vascular disease *n* = 446

Additional symptomatic information is available for 463 FSHD1 patients registered, 17.28 % (*n* = 80) reported hearing loss, evenly split across genders (46.25 % female; 53.75 % male) with 38.57 % (*n* = 27/70) reporting hearing loss before the age of 40 years. Retinal vascular disease is reported in just 10 patients 6 males and 4 females, with a mean age of 45.6 years (range 25–69).

A total of 9.85 % (*n* = 46) patients report having undergone scapular fixation surgery, 27 (58.69 %) of them had the procedure on both shoulders. The first operation has been performed at a mean age of 28.82, with 56 % having the surgery before age 30 years.

Thirty-seven (7.9 %) patients 22 males and 15 females, with a mean age of 50.96 years (range 20–76) reported the use of ventilation, with five (13.5 %) describing this as full time ventilation. Thirty-three of these (89.2 %) describe their current best motor function as requiring at least some assistance when walking.

A more in depth analysis of the clinical data will be discussed in a subsequent study.

## Discussion

### Successes and limitations

The UK FSHD patient registry contains information on over 518 FSHD patients, 475 of whom are confirmed to have FSHD1. Given the published FSHD1 prevalence of 3.95 FSHD1 patients per 100,000 in North East England [1] and based on a population of 64.1 million in UK in 2014, this would equal 31 % of population coverage. The primary goal of the registry is not to capture all patients within the UK but rather to identify patients who are interested in participating in research. This should be considered when drawing clinical conclusions as the registry is likely to represent a biased sample of the more able and willing patients or arguably could also represent the more severely affected that are more engaged to enrol. FSHD1 is the most common form of the disease with approximately 95 % of patients falling into this category, and this is similar in the UK registry population (91.7 %). The remaining 5 % are considered as FSHD2 patients [16] similar to the 8.3 % of the UK registry population. It will be interesting to assess whether there will be an increase of FSHD2 diagnosis and registration following the relatively recent development of confirmatory genetic testing for FSHD2.

Most of the patients registered are ambulant and although this could be due to a recruitment bias, when we compare our data with population based collected data the percentage of ambulant patients is similar, we therefore believe this self-reported data transmits and accurate snapshot of the general FSHD population [17–19]. The same is true when we look at the first location of muscle weakness, with facial and shoulder girdle being the most common locations. As already reported in the literature, distal or proximal lower limb involvement as the first manifestation is not unusual. In a previous report the frequency was 13 % for foot drop and 7 % for proximal lower limbs [20], the percentage is lower than in our population as they were considered as isolated manifestations and while in our cases the patients reported simultaneous locations for the beginning of the symptoms. An interesting aspect is the high incidence of hearing loss either under or above the age of 40. Further study is needed to determine whether this self-reported problem is in fact associated with any greater degree of hearing loss within the general population. From the recently published evidence-based guidelines from the American Academy of Neurology (AAN) [21] the recommendation regarding hearing loss is to screen patients with childhood onset as the prevalence of clinically relevant hearing loss is not clear in the general population. Respiratory involvement in FSHD is considered rare. From the literature and according to the guidelines of the AAN evidence from 2 studies suggests that respiratory involvement with an estimated frequency from 1.25 to 13 % and once more the self-reported respiratory status in our population is in accordance with this.

In asking the patients to provide the majority of the data through electronic communication, it has been possible to identify a large number of patients with FSHD in the UK in a short time frame and limited resources. A large amount of accurate and complete data now exists within the registry and is available to support the research community. In addition, there is a cohort of patients interested and able to participate in future clinical research. However, it should be noted the data provided are subjective and are not a substitute for data collected in a natural history study or clinical trial collected under GCP guidelines.

The input of detailed genetic data was delayed by the limited time and resources available to consultants within the NHS. This has been greatly increased through collaboration with diagnostic laboratories in the UK (Northern Genetics Service and Bristol Genetics Laboratory) that are able to provide details directly to the registry, as covered by the informed consent. In order to increase the coverage of genetic diagnosis further we will explore ways to receive this data directly from patients.

The registry has achieved its primary aim of helping to facilitate and accelerate recruitment into trials, evidenced by the infantile onset study mentioned above. The use of the registry led to the UK site (John Walton Muscular Dystrophy Research Centre) recruiting 70 % more patients than would have been available through local contacts alone. This significant increase is particularly important in rare diseases. Furthermore the response rates (>80 %) to the additional research questionnaires show that the utility of the registry goes above and beyond its primary purpose. The registry continues to consult with researchers in the UK and globally to ensure its use in clinical research, furthermore it could be utilised to develop and disseminate standards of care.

For the last few years, the European Union policy on Rare Diseases has stressed the need for an active collaboration in and between Member States as a way to promote equal access to care and to make the maximum use of the resources available for rare disease patients. The tools developed for this were at the level of the Member States the creation of National Plans for Rare Diseases and the nomination of Centres of Reference/Expertise; at a European level the future constitution of European Reference Networks are meant to help to fulfil that aim. For these instruments to be of use; data sharing and accurate, well established registries like the present one are an upmost requirement.

The registry has a mean recruitment rate of 21 registrations per month. A number of other registries utilise the same dual reporting system and have achieved similar rates, when taking into account different disease prevalence rates. The UK myotonic dystrophy registry (http://www.dm-registry.org/uk) has a mean rate of 16 per month while the incidence of myotonic dystrophy in the UK is thought to be higher than that of FSHD [1]. The Global FKRP registry (http://www.fkrp-registry.org/uk) collecting information about people with mutations in the FKRP gene has a lower rate of recruitment at an average of seven people joining per month, this is a much rarer condition (about 1 in 100,000 [1, 22]) however has global coverage. The persistent rate of inclusion seen in the UK FSHD registry suggests that there is an engaged and motivated FSHD population in the UK interested in participating and being informed about research.

### Future considerations

The registry now contains a wealth of data on a subset of the UK FSHD population; the next steps are to carry out an in depth analysis on this data, in particular looking at the progression of the condition. The registry allows for the collection and analysis of longitudinal data, and that analysis, particularly in relation to pain and quality of life may provide an interesting resource to improve care of FSHD patients in the UK. The registry will also be able to inform on genotype phenotype correlation for FSHD1 and FSHD2.

A sub study is planned using the information collected on scapular fixation, contacting the surgeons who performed the procedure to create a more in depth picture of how this operation is carried out in the UK. We hope this coupled with the detailed patient experience could help clinicians better inform patients considering this procedure in the future. Further analysis will also be carried out on the remaining clinical data to generate hypothesis for future research which may improve standards of care.

The UK FSHD patient registry contains all of the mandatory and highly encouraged items outlined in an internationally agreed dataset [13]. This data set is known to be collected by 15 registries either established or in the setup stage (Italy, Croatia, Netherlands, Ukraine, Spain, Argentina, Chine, Czech Republic, Georgia, UK, USA, Canada, Australia, Egypt and New Zealand) under the TREAT-NMD umbrella. This comparability could allow the creation of a Global FSHD registry, something TREAT-NMD has achieved successfully for paediatric neuromuscular diseases Duchenne muscular dystrophy and spinal muscular atrophy [23, 24]. The UK FSHD Registry would welcome any efforts of this kind from TREAT-NMD and would hope to be at the forefront of such developments. Furthermore there is an increased movement towards harmonisation of data among the wider rare disease community, not only with registries but also considering biobank, omics and imaging information. The UK FSHD registry has been working with RD-Connect as it pilots a number of initiatives working towards this goal [17–20].

## Electronic supplementary material

Below is the link to the electronic supplementary material.

Supplementary material 1 (DOCX 14 kb)

Supplementary material 2 (PDF 50 kb)

Supplementary material 3 (PDF 133 kb)

Supplementary material 4 (PDF 233 kb)

Supplementary material 5 (PDF 104 kb)
