# Supplementary material for: Design, set-up and utility of the UK facioscapulohumeral muscular dystrophy patient registry
Source: J Neurol. 2016 May 9;263:1401–8. doi: 10.1007/s00415-016-8132-1 (PMC4929161; doi:10.1007/s00415-016-8132-1)
Supplement: Supplementary file 1 — Supplementary material 1 (DOCX 14 kb) [file 415_2016_8132_MOESM1_ESM.docx]

|  | **All (n= 484)** | **FSHD 1 (n= 475)** | **FSHD 2 (n = 9)** |
| --- | --- | --- | --- |
| Details of genetic diagnosis available | 309 | 307 | 2 |
| Mean Age | 47.82 +/- 16.08 | 47.7 +/- 16.13 | 50 +/- 13.85 |
| Males/Females | 243/241 | 239/236 | 5/4 |

Online supplementary material Table: Summary of demographics in the UK FSHD Patient Registry.
