# Supplementary material for: Design, set-up and utility of the UK facioscapulohumeral muscular dystrophy patient registry
Source: J Neurol. 2016 May 9;263:1401–8. doi: 10.1007/s00415-016-8132-1 (PMC4929161; doi:10.1007/s00415-016-8132-1)
Supplement: Supplementary file 2 — Supplementary material 2 (PDF 50 kb) [file 415_2016_8132_MOESM2_ESM.pdf]

Muscular Dystrophy Campaign | TREAT-NMD Neuromuscular Network | MRC Medical Research Council

CONSENT FORM for Patients

UK Facioscapulohumeral Dystrophy Registry

Name of Researcher:

Prof Hanns Lochmüller
Experimental Chair of Myology
Institute of Genetic Medicine
Newcastle University

Please initial all boxes

1. I confirm that I have read and understand the information sheet dated January 2013 (version 1.0) for the above study. I have had the opportunity to consider the information, ask questions and have had these answered satisfactorily. ☐
2. I understand that my participation is voluntary and that I am free to withdraw at any time without giving any reason, without my medical care or legal rights being affected. ☐
3. By signing this document, I understand that I give consent for the storage of data on myself in the UK FSHD Patient Registry ☐
4. I understand that the storing of data will allow contact to be made with me if a suitable clinical trial becomes available ☐
5. However, I accept that allowing my data to be stored on this database does not mean I will automatically be entered into future clinical trials. ☐
6. I understand that the data I provide may be used to inform and plan future research. ☐
7. I understand that the results from future research may not have any direct implications for myself or my family. ☐
8. I confirm I am happy for specialists involved in my medical care to add relevant information to my database entry on my behalf. ☐
9. I am happy to consent to be included in this registry. ☐

Please sign below:

Name of Participant	Date	Signature
Name of Person taking consent	Date	Signature

Consent form date of issue: **April 2013**
Consent form version number: **2.0**
