# Supplementary material for: Design, set-up and utility of the UK facioscapulohumeral muscular dystrophy patient registry
Source: J Neurol. 2016 May 9;263:1401–8. doi: 10.1007/s00415-016-8132-1 (PMC4929161; doi:10.1007/s00415-016-8132-1)
Supplement: Supplementary file 3 — Supplementary material 3 (PDF 133 kb) [file 415_2016_8132_MOESM3_ESM.pdf]

UK FSHD Patient Registry

Steering Committee

Terms of Reference

UK FSHD Registry Steering Committee

Terms of Reference

1. A committee is hereby established as the FSHD (FSHD) Registry Steering Committee.
2. The following constitutes the terms of reference of the FSHD Registry Steering Committee.
3. In these terms of reference SC refers to the FSHD Registry Steering Committee.
4. The FSHD Registry has signed and adheres to the TREAT-NMD Charter, which therefore applies to this SC.
5. The SC can ask for advice from TREAT-NMD Project Ethics Council (PEC).

Chair and core members of the steering committee

6. The chair of the SC will be re-selected / elected annually by the SC members.
7. The core committee shall have at least one representative from clinical, genetic, research, TREAT-NMD, FSHD patient organisations, and FSHD patients and families at all times.
8. The membership of the core committee shall be restricted to 18 people.
9. One third of the core members of the SC will retire by rotation every 3 years but may offer themselves for re-election immediately for a further term; Members will be replaced like for like so as to maintain the correct composition.
10. Other organisations / groups / individuals that may have an interest in the development of the FSHD Registry may be invited to join the committee at any time with the agreement of the SC members.
11. The members of the SC have provided their services voluntarily and as such can withdraw from the steering committee at any point.
12. All SC members will be asked to complete a declaration of all potential conflicts of interest, which will be a public document that will be publishable on the TREAT-NMD website.
13. The SC gives permission for a list of all core members, and their professional profile, to be published on the website.

Meetings

14. Meetings will be held in person, by teleconference or by e-communication at least once per year, and upon request.
15. The venue for each meeting will be decided by the committee and will, in as far as possible, reflect the membership of the committee and the geographical remit. A suitable central venue might be used or venues might be changed to reflect full geographic spread.
16. Committee members will be reimbursed by the FSHD Registry for all travel expenses incurred for each meeting attended.

Records

17. A record of each meeting will be kept by an individual designated by the FSHD Registry to act as an administrator for the SC. It is the responsibility of the administrator to ensure that each

member of the committee receives the notes from the previous meeting within 2 weeks following each meeting.

Purpose

18. The purpose of the committee is to:

- Oversee all UK* enquiries to the FSHD Registry, providing approval, where applicable, for enquiries received; SC to decide whether enquiries are in-line with the interests of the FSHD Registry.

  **International (multi-country) enquiries will be approved by the TREAT-NMD Global Database Oversight Committee (TGDOC).*
- Approve trial/study recruitment enquiries and provide appropriate quality control (QC) for validation of the data before contacting patients; QC person to be appointed by the FSHD Registry and a contract and fee agreed, dependent upon the workload.
- Assess who will need to review the feasibility enquiry reports, for example the SC.
- Approve the necessary feasibility enquiry reports before the results are made available for the requesting party - dependent upon the enquiry.
- Approve all contracts made with industry/researchers.
- Approve all confidentiality/non-disclosure agreements from industry.
- Approve any contracts made and to agree a fee for the work undertaken by a geneticist to enter patient's genetic data into the FSHD Registry.
- Promote the FSHD Registry where applicable.

19. In pursuing this purpose the SC will ensure that the FSHD Registry is well governed.

20. Amendments of these terms of reference may be made at any time with the agreement of the committee.

Timescales

21. The SC will review its purpose, aims, objectives and achievements annually.

Non-liability

22. SC members will not be held liable for any problems encountered.

Voting

23. A minimum of two thirds of the SC must be present for a vote to occur and of those present, two thirds must be in favour for the vote to stand; voting can be done by email or at face-to-face meetings.
