# Supplementary material for: Design, set-up and utility of the UK facioscapulohumeral muscular dystrophy patient registry
Source: J Neurol. 2016 May 9;263:1401–8. doi: 10.1007/s00415-016-8132-1 (PMC4929161; doi:10.1007/s00415-016-8132-1)
Supplement: Supplementary file 4 — Supplementary material 4 (PDF 233 kb) [file 415_2016_8132_MOESM4_ESM.pdf]

UK FSHD Patient Registry

Standard Operating Procedures for National Registry Enquiries

FSHDFSHD Registry

Standard Operating Procedures

The FSHD (FSHD) Registry standard operating procedures for national registry enquiries were approved by the FSHD Registry 'Steering Committee'.

Procedure for a national registry enquiry from industry/researcher

A national registry enquiry is an enquiry into the FSHD Registry database for all eligible patients, who reside in the United Kingdom, according to the enquirer's inclusion criteria.

1. The requesting party (industry/researcher) will complete the feasibility questionnaire and provide details of the in-/exclusion criteria (**Appendix 1**).
2. The feasibility questionnaire will be checked by the FSHD Registry steering committee (SC) and a response will be provided within a minimum of 2 weeks of receiving the form (or an agreed timescale).
3. The feasibility questionnaire will be sent by the FSHD Registry curator (curator), by email to SC members for approval and comments. The curator will then collate replies from SC members and respond accordingly to the requesting party; approval is required from a minimum of two thirds of the SC.
4. Approval of the enquiry will form the basis of a contract between the FSHD Registry and the requesting party, which may include payment.
5. A contract is drawn up between the FSHD Registry and the requesting party, including the applicable fee and timescales (**Appendix 2**).
6. If a confidentiality/non-disclosure agreement is required, this should be requested before the feasibility questionnaire is completed.
7. If the enquiry is approved by the SC, the SC will indicate whether the feasibility report should be reviewed. The report will be provided to the requesting party within a minimum of 28 days once approval is obtained, dependent upon the enquiry. Specific timescale will be agreed with each requesting party.
8. A report of the feasibility results is drafted, and if necessary reviewed, before being made available to the requesting party; the FSHD registry will appoint the necessary people for this review.
9. For feasibility studies only, the clinical and genetic data stored in the registry will be assumed correct.
10. Upon verification of the clinical and genetic data those patients identified as eligible will be notified in writing and given the information about the upcoming clinical trial (**Appendix 3**).
11. The FSHD Registry should contact their patients and families to inform them of a clinical trial by providing them with the information in an appropriate language (**Appendix 4**).
12. The FSHD Registry should only in exceptional circumstances contact patients by phone to inform them of the clinical trials (potentially not "neutral" enough).

13. The FSHD Registry should provide general information to the major neuromuscular centres in the UK, and clinicians that participate in the registry, about the upcoming clinical trial as potentially eligible patients will be directed to their clinician to answer any questions they may have (**Appendix 5**).

14. The FSHD Registry should provide general information to all other patients registered about the upcoming clinical trial (**Appendix 6**).

15. A letter should be sent out to all patients registered informing them that the recruitment process is closed (**Appendix 7**).

16. When patients are contacted about a clinical trial they will be asked to contact their local trial centre for more information and to also inform them if they wish to participate.

17. Patient’s personal data will not be given out to third parties under any circumstances. This includes international enquiries handled by TREAT-NMD; see TREAT-NMD Charter (**Appendix 8**).

18. TREAT-NMD will deal with all international enquiries themselves; the FSHD Registry will be informed of any international enquiries that come through TREAT-NMD and may withdraw from the enquiry at this point.

19. Any international enquiries which are received by the FSHD Registry will be directed towards TREAT-NMD.

Procedure for entering and verifying clinical and genetic data

20. A patient’s clinical and genetic data is entered into the FSHD Registry by the patient’s regular clinician once the consent form has been received by the curator and a request email sent to the appropriate clinician.

21. After the feasibility study has been carried out and the requesting party wishes to recruit patients into the clinical trial, the data from the FSHD Registry will be attempted to be verified to ensure that it is correct before any patients are contacted.

22. If verification of the original clinical data is required then it will be carried out by an appropriate quality control (QC) person; The QC person will be appointed by the FSHD Registry and a contract and fee may be agreed, dependent upon the workload.

Transferring data to the TREAT-NMD Global Database (see TREAT-NMD Charter)

23. Transferred data will be reversibly encrypted and will include a standard and harmonised set of core data items (**Appendix 9**).

24. The FSHD Registry remains owner of the data included in the FSHD Registry and are free to grant access, re-utilization or permit extraction or grant any right in accordance to this ownership.

Liability

25. The FSHD Registry will be held liable for any problems encountered, in particular during a feasibility enquiry and the recruitment process.

26. The requesting party running the clinical trial will be held liable for any problems encountered during the trial process; the FSHD Registry does not endorse any clinical trial.

Reporting

27. The SC should submit an annual report to the FSHD Registry.

28. The SC chair and the curator will compile the report.
