# Supplementary material for: Design, set-up and utility of the UK facioscapulohumeral muscular dystrophy patient registry
Source: J Neurol. 2016 May 9;263:1401–8. doi: 10.1007/s00415-016-8132-1 (PMC4929161; doi:10.1007/s00415-016-8132-1)
Supplement: Supplementary file 5 — Supplementary material 5 (PDF 104 kb) [file 415_2016_8132_MOESM5_ESM.pdf]

April 2015
Version 1.0

Costing Guidelines for UK FSHD Registry

The UK FSHD is entirely supported by non-profit funding form patient organisations in the United Kingdom. For sustainability it is necessary that fees are charged in exchange for the services that can be provided.

The UK FSHD Registry supports and encourages applications from researchers (commercial and non-commercial) working to improve knowledge, understanding, care and treatment of FSHD. Research which may benefit from interactions with the registry includes but is not limited to:

- Clinical research that will improve standards of care and best practices
- Scientific research that increases understanding of disease mechanism and pathology
- Epidemiological studies to better understand the prevalence and progression of the condition.
- Clinical trials of new therapies and treatments.

Definitions

Registry Service:
Actions that are routinely carried out as part of the Registry's services:

- Feasibility enquiry, including data export, analysis and generation of a report.
- Inform (via e-mail or post) registrants and associated professionals about a study
- Custom data collection (though online or paper based questionnaires)
- Coordination of custom data collections.

Non-commercial enquires

There is no cost associated with the use of the registry for non-commercial organisations. Non-commercial organisations include but are not limited to academic institutions, patient organisations and government agencies.

Exceptions

Non-commercial organisations who contact us at the point of grant writing.
We encourage this in all cases and we are able to provide feasibility data to help in the grant writing free of charge. We would then request some funds towards the Registry to be included in the grant application where appropriate. This would be costed by Newcastle University on a cost recovery basis only.

Disclaimer: These are guidelines only and each enquiry will be assessed on a case by case basis.

April 2015
Version 1.0

Commercial Enquires

Fees will be charged for commercial enquires including those from pharmaceutical and biotechnology companies. An outline of the fees charged are summarised in the table below. This is just a guide and every enquiry will be assessed on case by case basis.

Exceptions

A commercial partner wishing to form an ongoing collaboration with the Registry.
In the interest of future collaboration we may provide feasibility data to a commercial partner free of charge. If the collaboration moves into a recruitment phase the costs for producing the feasibility report will be included in the recruitment costs.

Type of Activity	Fee Charged
Handling Fee	£500
**Feasibility Enquiry**	
Data pull and report generation	£1,500
Specialist data request	Assessed on a case by case basis.
**Recruitment Enquiry**	
Mail-out of study information (e-mail and/or post)	£2,500
**Questionnaire Study.**	
Questionnaire mail-out (e-mail and/or post)	£2,500
Questionnaire coordination and follow up (email and/or post)	£5,000
Questionnaire data collection.	£2,500
Questionnaire data analysis.	Offered on a case by case basis.
Questionnaire adaptation (online)	£1,000 per data set.
Amendment to Ethics	£1,000 per amendment.

Disclaimer: These are guidelines only and each enquiry will be assessed on a case by case basis.

Examples:

Enquiry 1:

A pharmaceutical company would like a report including the total numbers of patients in the UK, and the numbers of ambulant and non-ambulant patients in a 50 mile radius of Manchester.

Handling fee: £500
Data Pull and report generation: £1,500
Total cost: £2,000.

Enquiry 2:
A commercial company would like to send a link to a questionnaire to all patients on the registry would like us to coordinate follow up and send paper copies to those who have not completed online. The online questionnaire should be set up by the Registry; this requires an amendment to the ethical approval.

Handling fee: £500
Questionnaire mail-out (e-mail): £2,500
Questionnaire coordination and follow up (e-mail and post): £5,000
Questionnaire adaptation (online): £1,000
Amendment to Ethics: £1,000
Total cost: £10,000

Enquiry 3:
A pharmaceutical company would like information to be sent via e-mail to all ambulant patients over 18 about a study. They do not require additional follow up.

Handling fee: £500
Mail-out of study information (e-mail): £2,500
Total cost to industry: £3,000
